# Burkitt Lymphoma: Pathogenesis and Immune Evasion

**DOI:** 10.1155/2010/516047

**Published:** 2010-10-05

**Authors:** Jason M. God, Azizul Haque

**Affiliations:** Department of Microbiology and Immunology, Charles Darby Children's Research Institute, Hollings Cancer Center, Medical University of South Carolina, 173 Ashley Avenue, Charleston, SC 29425, USA

## Abstract

B-cell lymphomas arise at distinct stages of cellular development and maturation, potentially influencing antigen (Ag) presentation and T-cell recognition. Burkitt lymphoma (BL) is a highly malignant B-cell tumor associated with Epstein-Barr Virus (EBV) infection. Although BL can be effectively treated in adults and children, leading to high survival rates, its ability to mask itself from the immune system makes BL an intriguing disease to study. In this paper, we will provide an overview of BL and its association with EBV and the *c-myc* oncogene. The contributions of EBV and *c-myc* to B-cell transformation, proliferation, or attenuation of cellular network and immune recognition or evasion will be summarized. We will also discuss the various pathways by which BL escapes immune detection by inhibiting both HLA class I- and II-mediated Ag presentation to T cells. Finally, we will provide an overview of recent developments suggesting the existence of BL-associated inhibitory molecules that may block HLA class II-mediated Ag presentation to CD4+ T cells, facilitating immune escape of BL.

## 1. Introduction

Burkitt Lymphoma (BL) is a high-grade B-cell malignancy occurring most frequently in children in areas with holoendemic and hyperendemic malaria, and with lesser frequency in all other parts of the world [1, 2]. This aggressive neoplasm is classified as a Non-Hodgkin's Lymphoma (NHL) and has the fastest doubling time among human tumors [3]. BL is subdivided into three different categories based on epidemiological observations: endemic BL (eBL), sporadic BL (sBL), and HIV-associated BL. About 95% of eBL cases are associated with Epstein-Barr Virus (EBV) and are commonly found in Equatorial Africa and Papua New Guinea where malarial diseases are highly prevalent. In contrast, only 5–15% of sBL and 40% of HIV-associated BL are EBV positive [4–6].

EBV is a member of the herpes family of double-stranded DNA viruses with an icosahedral-shaped capsid [7]. Worldwide more than 90% of all people become infected with EBV at some point during their lifetime [4, 8]. Though most infected individuals remain healthy, EBV is capable of leading to pathologic conditions, being linked to a variety of human diseases and malignancies. EBV also has the potential to transform normal human B lymphocytes into continuously growing immortalized cells such as BL and B-lymphoblastoid cells. It is present in approximately 50% of Hodgkin's Lymphoma (HL), a disease which accounts for 1% of all malignancies in the United States, and is found with varying frequency in NHL [9–11]. EBV is implicated in infectious mononucleosis, T-cell lymphoma, adult T-cell leukemia, Natural Killer cell (NK) leukemia, posttransplant lymphoproliferative disorder, nasopharyngeal carcinoma, and various other lymphoid and epithelial malignancies [12–16]. In most individuals, infection of B lymphocytes by EBV is followed by a cytotoxic CD8+ T cell (CTL) response that controls the spread of the virus. This response can be generated by latent viral proteins including EBNAs (EBV Nuclear Ags) and LMPs (Latent Membrane Proteins), but is dominantly targeted towards EBNA3; the LMPs also elicit a cytotoxic CD4+ T cell response to EBV-transformed B-cell lines [17]. T cells also recognize several lytic cycle proteins, such as BZLF1, BMLF1, BMRF1, and BHRF1 [18]. In spite of this vigorous CD8+ T cell response, a population of infected B cells escapes immune-mediated elimination. Immunodeficiencies resulting from certain genetic disorders, organ transplantation, or infectious diseases (e.g., AIDS, malaria) can lead to reactivation and outgrowth of these EBV-infected B cells [10]. EBV infection can also lead to the generation of a number of proteins (e.g., EBNA1, *c-myc*) which are involved in decreasing immune recognition of malignant B cells. In the following sections, we will look further at the role of EBV as a possible causal agent of BL as well as its involvement in contributing to immune evasion.

The common characteristic of virtually all BL is translocation of the *MYC* proto-oncogene to an immunoglobulin (Ig) locus [19]. *MYC* encodes the *c-myc *transcription factor which was first discovered nearly thirty years ago as a cellular homologue of an avian retroviral oncogene [20]. Since that time, *MYC *has been recognized as one of the most commonly activated oncogenes in human cancers. It is estimated to play a role in 20% of all cancers, with potential involvement in 100,000 cancer deaths in the US each year [21–23]. *c-myc *is a transcription factor belonging to the class of basic helix/loop/helix/leucine zipper proteins and high-throughput screening has indicated that 15% of all known genes lie within its target gene network [23, 24]. Functions of genes in this network include the regulation of cell-cycle progression, proliferation, differentiation, and apoptosis [23, 25]. Under normal conditions, *c-myc* is activated in response to mitogenic factors and repressed upon exposure to antiproliferative signals. The involvement of* c-myc* in leading to development of BL, as well as its role in decreasing immunogenicity, will be addressed later in this paper.

## 2. Burkitt Lymphoma

### 2.1. Overview

Studies suggest that eBL and sBL differ in geographical distribution and degree of association with EBV. eBL occurs primarily in equatorial Africa and Papua New Guinea, and has a 95% association with EBV. sBL, which accounts for 1%-2% of adult lymphomas and 30%–50% of pediatric lymphomas in the United States (US) and Western Europe, occurs in all other parts of the world, but has only a 15% association with EBV [4, 5, 26]. Subtypes of BL also differ in clinical manifestation. Typically, eBL presents as tumors affecting the jaw and facial bones while sBL more commonly arises in the gut and upper respiratory tract, forming tumors in the Waldeyer ring [27, 28]. HIV-associated BL characteristically involves the lymph nodes and bone marrow [19]. While eBL primarily affects children 4–7 y, sBL is seen in both children and young adults, having a median age of 30 y [3]. For all three types of BL, males are more commonly affected than females [3]. For the years 1973–2005, there were 3,058 cases of BL diagnosed in the US. Five-year mortality showed a positive correlation to age with pediatric cases having a ~25% mortality, adult cases 50% and geriatric cases 70% [29]. 

A feature observed in nearly 100% of BL is a reciprocal chromosomal translocation involving the proto-oncogene *MYC* on chromosome 8 and one of the Ig gene heavy or light chain loci on chromosomes 14, 2, or 22 [19]. The translocation to an Ig locus leads to deregulation and constitutive expression of c-*myc*, with an overall effect of uncontrolled proliferation as well as a reduced threshold for induction of apoptosis [24]. Characteristics of BL cells seem to point to a germinal center (GC) origin. BL cells phenotypically resemble centroblasts, expressing high levels of BCL-6 [30] and show signs of somatic hypermutation (SHM), a common characteristic of GC B cells [31, 32]. A final piece of evidence that indicates a GC origin stems from the *MYC *translocations: the chromosome breakpoints involved in these translocations suggest a mistake of class-switch recombination (CSR) or SHM, two processes that occur in GC B cells [33, 34]. *MYC *translocation is considered a hallmark for BL and its role in disease progression and immune evasion will be discussed in greater detail later in this paper.

EBV-positive BL cells express low levels of activation markers, adhesion molecules, and costimulatory molecules and they grow as single-cell suspensions, rather than in clumps typical of B-lymphoblastoid cells [35]. Unlike B-lymphoblastoid cell lines (B-LCL), BL cells exhibit a deficiency in stimulation of CD8+ T cells via HLA class I molecules [36]. We have shown that BL cells express detectable levels of HLA class II, but fail to effectively stimulate CD4+ T cells [37]. HLA class II proteins on BL cells were capable of binding antigenic peptides but the class II-peptide complexes were not functional (unpublished data). However, under acidic conditions (pH = 5.5), BL cells were capable of forming functional class II-peptide complexes that could stimulate T cells at neutral pH. Acidic eluates obtained from BL cells diminished functional HLA class II Ag presentation by B-LCL and CD4+ T cell responses under physiological condition [37], suggesting that BL-associated inhibitory molecules (BLAIM) may perturb CD4+ T cell recognition of BL cells. It remains unclear how BLAIM could interact with class II and the T-cell receptor (TcR), disrupting HLA class II-restricted immune recognition of BL.

### 2.2. HIV and Malaria as Coinfections

Study suggests that the geographical distribution of eBL corresponds greatly to that of malarial diseases and that the prevention of malaria may lead to a decreased incidence of eBL [38]. For eBL, there is a strong association with endemic *Plasmodium falciparum* malaria. A study conducted in Malawi (which is endemic for both malaria and BL) revealed that children expressing high levels of antibodies for both pathogens had 13 times the risk of developing eBL when compared to children with low antibody levels [38]. Additionally, children with malarial diseases living in areas endemic for both EBV and malaria were shown to have significantly higher levels of EBV antibodies than either their healthy counterparts or children living in areas of sBL [39–41]. In spite of their strong association, the relative functions of the malarial parasite and EBV in the development of BL remains elusive. It is generally believed that hyperstimulation of B cells and suppression of T-cell activity by malaria allow for reactivation of EBV in infected B cells, which consequently increase in numbers. Suppression of T-cell activity is suggested by the fact that children 5–9 years old living in areas holoendemic for malaria displayed inferior IFN-*γ* responses when compared to children living in malaria variable regions. This age range coincides with the peak age incidence for eBL [42]. Chene et al. [43] have reported that increasing levels of malarial Ags become trapped in secondary lymphoid organs, leading to hyperactivation of the germinal centers and increased SHM. As *MYC *translocations take place in the germinal centers during SHM, it is plausible to imagine an increase in the number of translocations which could ultimately lead to the development of BL. 

HIV represents another infection that may aggravate the pathogenesis of BL. Up to 20% of HIV-associated NHL in the Western World are BL, and HIV-positive patients are believed to have a 200–1000-fold greater risk of developing BL than HIV-negative patients [26]. It is widely accepted that immunodeficiency resulting from HIV infection is responsible for reactivation of EBV in latently infected B cells and this ultimately progresses to BL [38]. Recent studies have shown that the priming of circulating EBV-specific CD8+ T cells is dependent on CD4+ T cells [10, 44]. In HIV-infected individuals the CD4+ T-cell count is greatly reduced, leading to diminished CD8+ T-cell activity which permits reactivation of EBV-infected B cells [10]. While HIV-associated BL can be treated with various short-term, aggressive chemotherapeutic regimens in conjunction with highly active antiretroviral therapy (HAART), toxicity and immunosupression pose a threat to the patients. The use of rituximab in immunocompromised patients is also a debated issue, suggesting the need for the development of less toxic and more specific immunotherapies.

### 2.3. Treatment of BL

The evolution of chemotherapeutic treatments for BL, as well those currently in use, has been reviewed by Aldoss et al. [45] and should be considered in order to appreciate the need for improved therapies. While it is beyond the scope of this review to thoroughly describe these treatments, a few salient points can be addressed. Early treatments for BL sought to use chemotherapy regimens that were being used for other NHL. However, when used for BL, they showed far inferior responses and cure rates, and these results were attributed to BL's rapid doubling time. To overcome this, intensive chemotherapy with short intervals between treatments was explored as an option. These regimens showed improved responses, but increased treatment-associated toxicities. Additionally, adults (particularly the elderly) had inferior response rates and were less tolerant to the toxicities than children. Various chemotherapeutic treatment options currently exist for treatment of BL, but virtually all have the same shortcomings such as inferior responses and decreased tolerance to treatment-associated toxicities in adults. An additional confounding factor is coinfection with HIV, in which aggressive chemotherapy further aggravates their already immunodeficient state and leads to severe toxicities [3, 46–51].

Immunotherapeutic treatment of BL is found in the form of the anti-CD20 monoclonal antibody, rituximab, which induces cell death in B cells by cell-dependent cytotoxicity, antibody-dependent cell-mediated cytotoxicity, or complement activation. Rituximab used in conjunction with chemotherapy has led to increased response rates in BL patients [52]. However, toxicities from the chemotherapy are still an issue in many patients, and additional immunosuppression resulting from the use of rituximab is also a concern for HIV-infected BL patients. In a study by Oriol et al. [53], a clinical trial evaluated the use of chemoimmunotherapy (intensive chemotherapy with 8 doses of rituximab) in HIV-infected and HIV-negative adult BL patients. The complete remission rates for the 2 groups were very similar, but the HIV-infected patients experienced a higher incidence of severe mucositis and infections. More recently, the anti-CD22 monoclonal antibody, epratuzumab, has entered clinical trials and shows a synergistic effect when used concomitantly with rituximab [54]. Thus, chemotherapy and chemoimmunotherapy have proved themselves as invaluable tools in the treatment of BL. However, due to the inferior tolerance and responses observed in adults and HIV patients, there remains a need for more targeted and less toxic therapies for BL. Our study indicates that BLAIM impairs the HLA class II Ag presentation pathway. If this molecule can be identified and characterized, it may allow for the development of monoclonal antibodies which could have much more targeted effects than existing antibodies. This could also be combined with rituximab or epratuzumab to eliminate the need for toxic chemotherapies in some patients. In addition to the therapies discussed above for BL, adoptive transfer of EBV-specific T cells has shown some promise in treating hematopoietic stem cell transplant and solid organ transplant patients [55]. Any such therapy used for treatment of BL would have to address the low immunogenicity of EBNA1 as well as the decreased HLA class II response imparted by BLAIM that is implied by our research.

## 3. Burkitt Lymphoma and Transformation of B Cells

### 3.1. EBV and B-Cell Transformation

EBV is known to be associated with BL to varying degrees, but the exact role that it plays in the development of BL has remained elusive. It is known that EBV is capable of transforming B cells and this may play a role in the pathogenesis of BL. The entry of EBV into B cells involves at least five viral glycoproteins. EBV binding is partially mediated by the viral envelope protein gp350 which binds to complement receptor 2 (CR2) on B cells and tethers the virus to the B cell, allowing viral gp42 to bind to HLA class II proteins [56, 57]. Upon binding of EBV to the B cell, gp42 triggers membrane fusion which is carried out by the viral proteins gB, gH, and gL [58, 59].

 Following infection, EBV produces an array of Ags including six EBNAs, early Ags, viral capsid Ag, EBV-induced membrane Ag and latent membrane proteins (LMPs). The six EBNA proteins have varying expression patterns which relate to different pathologic conditions. EBV has three transcriptionally distinct forms of latency, each with a different expression pattern for latent EBV-encoded genes [34]. Latency I, associated with BL, is characterized by expression of EBNA1 and small noncoding EBV RNAs (EBERs). Latency II, associated with HL, is characterized by expression of EBNA1, LMP1, LMP2, and EBERs. Latency III, observed in posttransplant lymphoproliferative disorders, includes expression of all EBNAs, EBERs, and LMPs [34, 60, 61].

As it pertains to transformation of B cells, it seems the EBV products of primary importance are EBNA1, EBNA2, EBNA3C, EBNA-LP, LMP1, and LMP2A [62]. EBNA2 is one of the first viral protein to be expressed following infection of B cells and its expression is required for transformation [12, 63]. It works in concert with EBNA-LP to activate cyclin D2, driving the B cell from G_0_ into G_1_, and its inactivation results in cell-cycle arrest and entry into apoptosis [64, 65]. EBNA2's role in transformation is essential because it acts as a transactivator for all six EBNAs as well as the LMPs. EBNA2 also interacts with the cellular DNA-binding elements RBP-J*κ* and PU.1 to mediate the transcription of numerous cellular genes which contribute to transformation [63, 66]. Comprehensive screening has identified 550 cellular genes that are either significantly induced or repressed by EBNA2, including *MYC* [67, 68]. 

Among the viral genes under transcriptional control of EBNA2 are the transmembrane proteins LMP1 and LMP2A. These proteins mimic normal B-cell molecules and deliver growth and survival signals commonly seen in B-LCL. LMP1 functions as a homologue of CD40 and signals through the tumor-necrosis-factor-receptor-associated factors (TRAFs) that result in activation of the transcription factor NF-*κ*B, leading to cell survival and growth [69–71]. LMP2A performs a similar function by mimicking the signaling of the B-cell receptor (BCR). Both LMP2A and the BCR signal through immunoreceptor tyrosine-based activation motifs and trigger the B-cell signaling pathway that leads to production of inositol trisphosphate (IP_3_) and diacylglycerol (DAG), ultimately resulting in cell survival but not cell growth [34, 70]. In addition, EBNA3C has a role in cell cycle progression through degradation of the tumor suppressor protein, pRb [72].

### 3.2. *c-myc* and B-Cell Transformation

The translocation of *MYC *to an Ig locus is considered a hallmark of BL. The most frequently observed translocations show a break in the long arm of chromosome 8, adjacent to or within the *MYC *gene. 80% of all BL show a t(8:14) translocation while the other observed translocations are t(8:22) and t(8:2) [24, 73]. *MYC* translocation requires activation-induced cytidine deaminase (AID), leading to the thinking that BL arises from germinal center B cells, as AID is highly expressed in the germinal center and plays a role in SHM and CSR [24, 74, 75]. Due to the ambiguous role of EBV in eBL and sBL, the breakpoints involved in *MYC *translocation have been analyzed and suggest that those in eBL are more closely associated with the joining region while those in sBL are more closely associated with switch regions [3, 76–78]. Regardless of the particular translocation involved or the location of the breakpoint, all *MYC* translocations in BL bring the gene under control of an Ig locus, resulting in its constitutive activation. Because the expression of many genes is controlled by *c-myc*, the outcome of its constitutive expression is cell growth, uncontrolled proliferation, and a reduced apoptotic threshold [24]. Overexpression of *c-myc* contributes to proliferation by inducing the activity of cyclins, while at the same time repressing the activity of the cyclin inhibitor p27. The proapoptotic properties of *c-myc* involve both the extrinsic (through interactions with TRAIL) and intrinsic (through interactions with p53 and Bim) pathways [79]. When taken together, these properties explain the observation that BL cells have a high proliferative index while remaining susceptible to apoptosis [24, 80].

Even though translocation of *MYC *is generally considered a hallmark for BL and this characteristic has been uniformly observed across the different BL subtypes, *c-myc *does not act alone. Overexpression of *c-myc* drives cells into the cell cycle, but it also leads to apoptosis in the absence of apoptosis-inhibiting signals. These signals may be provided by other oncogenes, such as *ras* or *bcl-2*, or in the case of EBV-positive BL by EBNA1, which is antiapoptotic [81, 82]. Further, in order for *c-myc* to initiate its transcriptional activities, it must first form a heterodimer with the constitutively expressed *max* [82]. If the formation of *c-myc*/*max* heterodimers is prevented, cells do not undergo *c-myc*-induced transformation. Thus, while overexpression of *c-myc* is required for development of BL, it works in concert with other proteins to exert its effects on the cell. 

It should be noted that some studies have shown a very small number of BL cases do not show any *MYC* translocation, yet still overexpress *c-myc*. This has been demonstrated for <10% of sBL and linked to miRNA deregulation [83]. In cases of BL that are negative for *MYC *translocations, downregulation of the miRNAs let-7c and miRNA-34b (which negatively regulate *c-myc* mRNA translation) has been observed and postulated to be the cause of *c-myc* overexpression. In contrast, cases of BL with *MYC *translocations also show higher expression levels of these miRNAs. This gives two distinct mechanisms by which BL cells may come to overexpress *c-myc*: (i) the translocation of *MYC* to an Ig locus, and (ii) downregulation of miRNAs which regulate translation of *c-myc* mRNA [83].

## 4. Burkitt Lymphoma and Immune Evasion

### 4.1. EBNA1 in Immune Evasion of BL

The immune system is capable of generating both CD4+ and CD8+ T-cell responses to several latent and lytic phase EBV-associated Ags, such as LMP1, LMP2, EBNA2, and EBNA3. Unfortunately, BL cells generally express only the EBNA1 protein, which is poorly antigenic and has little to no HLA class I response. The mechanism by which EBNA1 escapes HLA class I presentation involves the presence of an internal glycine-alanine (gly-ala) repeat that has a dual role in this process (Table 1). First, the gly-ala repeat prevents the formation of a functional complex with the proteasome, thus blocking the protein degradation necessary for HLA class I loading and presentation to CD8+ T cells [84]. Secondly, the gly-ala repeat causes a decrease in the translation of the EBNA1 mRNA, reducing the production of antigenic peptides [85]. Because EBNA1 limits its presentation by HLA class I molecules, the CD8+ T cell response to BL is largely diminished. 

While EBNA1 limits its presentation via the HLA class I pathway, alternative methods of Ag presentation may allow for EBNA1 epitopes to be displayed by HLA class II proteins. Studies conducted in the lab of Christian Munz reveal that EBV-seropositive adults virtually always express EBNA1-specific CD4+ T cells [93, 94]. Further, Leung et al. [95] have demonstrated that autophagy may play a key role in the presentation of EBNA1 epitopes by HLA class II, which is normally thought of in the context of presenting extracellular Ags. In this study they demonstrated that removal of a nuclear location sequence (NLS) from EBNA1 resulted in HLA class II presentation of EBNA1 epitopes on the surface of B-LCL [95, 96]. Furthermore, the resulting Ag presentation was sensitive to treatment with 3-methyladenine, an inhibitor of autophagy. While these results certainly shed valuable light on the role of CD4+ T cells in recognizing the EBNA1 Ag and open the door to possible therapies for certain EBV-related disorders, the implications for BL may be more limited. Our recent study has also shown that BL cells are deficient in their ability to functionally present Ags via the HLA class II pathway. Thus, understanding the mechanisms by which BL cells escape immune recognition, may open new avenues for devising novel immunotherapies against malignant B cells.

### 4.2. *c-myc* in Immune Evasion of BL

The picture described above for EBNA1 is somewhat clouded with the realization that EBNA1 in B-LCL can be recognized by CD8+ T cells, implying that EBNA1 is not the sole player leading to immune evasion by BL, and other factors could be involved [91, 92]. EBNA1 is generally the only EBV Ag produced in EBV-positive BL, but atypical cases of BL do exist in which other EBV Ags are synthesized. The immune response to these tumors was addressed by Kelly et al. [97] in a study where 10 cell lines were established from confirmed eBL tumors. Screening of the cell lines revealed 3 which expressed EBNA1, -3A, -3B, 3C, and truncated LMP. As previously discussed, the EBNA3 family is highly immunogenic and a better target for inducing CD8+ T-cell responses. Yet, even in these atypical BL tumors which expressed the EBNA3 family, optimum CD8+ T cell activation was not observed.

These puzzling observations can be partially explained by *c-myc's *activity in reducing the immunogenicity of BL. It is well established that cytotoxic CD8+ T cells do not efficiently recognize BL cells (whether positive or negative for EBV) and are thus incapable of mounting an immune response [35]. *c-myc* expression in some tumors is inversely correlated with expression of HLA class I, and this may also be true for BL (Table 1). In this study, though the mechanisms responsible were not elucidated, an immunogenic B-LCL that is normally recognized by cytotoxic T cells was rendered nonimmunogenic when *c-myc* was overexpressed [35]. An additional piece of the puzzle in determining how *c-myc* exerts its immune inhibitory activities was provided when Schlee et al. [86] demonstrated that *c-myc* overexpression alters mRNA profiles in conditional B-cell lymphoma lines via the NF*κ*B activation pathway (Table 1).

Earlier studies had shown that overexpression of *c-myc *led to decreased expression of accessory molecules important in the immune response, including LFA-1, LFA-3, ICAM-1, and TAP [87–90] (Table 1). Conversely, Staege et al. [35] found that inactivation of *c-myc* results in restored expression levels of these accessory molecules and Ag presentation. Further insight into *c-myc's* involvement in immune evasion is gleaned from studies showing that another hallmark feature of BL is little to no expression of NF-*κ*B [90, 98, 99]. It was eventually determined that low NF-*κ*B was responsible for the decreased expression of the accessory molecules observed in conditions of *c-myc* overexpression. In BL, NF-*κ*B regulates proapoptotic genes and restoration of its expression results in increased expression of Fas nd eventual cell death [90]. 

 In addition to the impaired NF*κ*B response, Schlee et al. [86] also found that genes involved in interferon (IFN) responses were down-regulated in BL when compared to B-LCL. These genes included STAT1 and STAT2 and a similar pattern was observed for genes connected to B-cell receptor signaling. This group also showed a sharp decrease in STAT1 protein and RNA expression in BL cells relative to B-LCL. The negative regulation of STAT1 by *c-myc* occurred directly, through blocking STAT1 expression, and indirectly by suppressing IFN induction. Thus, the overexpression of *c-myc *appears capable of decreasing the immunogenicity of both EBV-negative and EBV-positive BL by altering genes in the NF-*κ*B pathway. In EBV-positive BL cells, these activities would augment the poor antigenic property of EBNA1, facilitating immune evasion.

### 4.3. Evasion of HLA Class I Presentation

**Figure 1:**
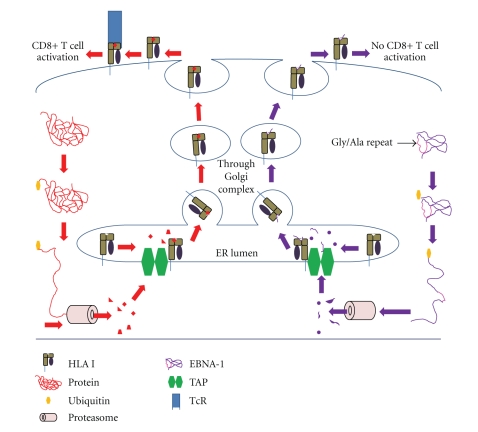
Defects in HLA class I antigen processing and presentation associated with BL. Cytosolic proteins are ubiquitinated, marking them for proteasomal degradation. Peptides generated from proteasomal degradation are then transported into the endoplasmic reticulum (ER) lumen by TAP (transporter associated with Ag presentation) and loaded onto HLA class I proteins. These class I peptide complexes are transported through the Golgi network to the cell surface for presentation to CD8+ T cells. The EBV EBNA1 protein contains a Gly/Ala repeat that impairs its proteasomal processing, resulting in the generation of peptides that are not readily accessible to class I molecules. Thus, peptides generated from EBNA1 proteins are unable to activate CD8+ T cells in the context of HLA class I molecules.

HLA class I molecules are expressed on every nucleated cell of the body and are involved in Ag presentation of cytosolic peptides to CD8+ T cells [100, 101]. As proteins are produced in the cytosol, they may become ubiquitinated, marking them for degradation in the proteasome (Figure 1). Peptides resulting from proteasomal degradation are transported into the lumen of the endoplasmic reticulum (ER) via the transporter associated with Ag presentation (TAP). In the ER lumen, HLA class I molecules can bind peptides approximately 8–10 amino acids long. Peptide-HLA class I complexes are then transported to the cell membrane for presentation to CTL (Figure 1). If the peptide is recognized by a CTL as being non-self, the CTL may induce apoptosis in the target cell through the perforin/granzyme pathway. Viral proteins are synthesized in the cytosol and are subjected to the same proteasomal degradation and HLA class I presentation for cellular protein as shown in Figure 1. This process is essential to the immune system's ability to monitor for viral infections and transformed cells and to mount an appropriate response. Indeed, the importance of this pathway is revealed by the strategy of HLA class I downregulation employed by many viruses and transformed cells to reduce their immunogenicity. 

Although the pathway described above is the primary mechanism by which cells present endogenous Ag in the context of HLA class I, there are two alternative strategies that allow cells to present exogenous Ags via HLA class I molecules. First, HLA class I molecules may bind short exogenous peptides for presentation to CTL [102]. Studies have demonstrated that some exogenous Ags can be directly delivered to the ER and that these Ags can be presented by HLA class I molecules [103, 104]. In spite of these mechanisms by which Ag may be presented by HLA class I, BL cells are not effectively controlled by CTL [84, 105]. EBNA1, the sole EBV Ag expressed in EBV-positive BL, uses an internal gly-ala repeat to prevent its optimum presentation by HLA class I and largely escapes CTL detection (Figure 1). 

A more general approach is also utilized by BL cells to avoid detection by CTL, and this involves the downregulation of HLA class I protein expression (Table 1). This is a common strategy that is widely observed in various virus-infected and transformed cells. In one study, BL lines derived from five HLA-A11-positive donors (both EBV positive and negative) were shown to have decreased expression levels of HLA-A11 and were resistant to lysis by HLA-A11-restricted CTLs generated by stimulation with autologous B-LCL [106]. Other groups have investigated this aspect of BL immune evasion and reported similar observations, with HLA-A11 being the most commonly reported down-regulated form of HLA class I molecules [107, 108]. Based on the observations that a CTL response is capable of controlling outgrowth of B-LCL but is ineffective against EBV-positive BL cells, Jilg et al. [109] found that B-LCL expressed considerably higher levels of HLA class I molecules than those of BL. By preventing HLA class I presentation of the lone EBV Ag expressed in BL and by down-regulating HLA class I expression, BL minimizes detection by CTL and thus escapes a major portion of the immune response to both EBV-positive and EBV-negative BL. With CTL detection being largely avoided, the job of immune detection is left up to HLA class II-mediated Ag presentation.

### 4.4. Evasion of HLA Class II Presentation

While the HLA class I-mediated immune response to BL has been very well studied, the HLA class II-mediated response has not received nearly as much attention. BL cells express HLA class II molecules but their role in optimum Ag presentation and CD4+ T-cell stimulation remains unclear. HLA class II proteins are constitutively expressed by professional Ag presenting cells (APCs), such as macrophages, dendritic cells, and B cells [110, 111]. Unlike the HLA class I binding pocket, the ends of the class II peptide-binding groove are open, thus allowing the bound peptides to extend from the ends of the groove, accommodating larger peptides of approximately 12–25 amino acids in length [112–114]. Additionally, HLA class I primarily presents endogenous Ags while HLA class II presents exogenous Ags including tumor and viral Ags. Although the emphasis has been given to the generation of CTL responses, these efforts have only led to short and weak responses in vaccinated patients. Increasing evidence suggests that the induction of optimal antitumor immunity requires both CD4+ and CD8+ T cells specific for tumor-associated Ags [115, 116].

**Figure 2:**
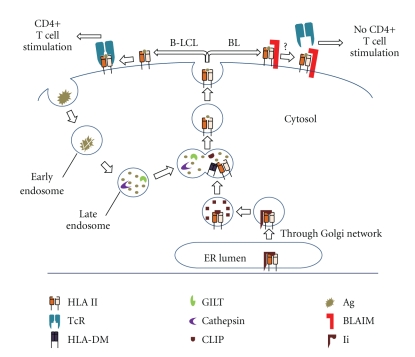
Defects in HLA class II antigen processing and presentation associated with BL. Extracellular Ags are endocytosed and degraded in increasingly acidified endolysosomal compartments. GILT helps in Ag/peptide processing by reducing disulfide bonds in the acidic environment. These peptides are further processed by acidic cathepsins for loading onto HLA class II proteins. HLA class II is synthesized in the ER lumen and forms a complex with Ii that is transported through the trans-Golgi network for processing by cathepsins in the endolysosomal compartments. Here Ii is degraded, leaving a fragment, CLIP, in the class II binding groove. HLA-DM mediates the release of CLIP and the loading of appropriate peptides onto HLA class II molecules. These complexes are then transported to the cell surface for presentation to CD4+ T cells. BL-associated inhibitory molecules (BLAIM) may interfere with functional class II presentation that perturbs CD4+ T cell recognition of BL.

Numerous differences exist between HLA class I and class II synthesis and Ag processing, and these differences partially reflect the nature of the Ags bound by each class. HLA class II molecules are composed of alpha (*α*) and beta (*β*) chains which are assembled in the ER and associated with the invariant chain (Ii) [112–114]. Ii aids in transporting class II molecules to the endolysosomal compartments, where Ii is sequentially degraded by cathepsins, leaving class II-associated invariant-chain peptide (CLIP) on the class II binding groove [110, 113]. Ags/Peptides are also processed in the endolysosomal compartments by acidic cathepsins for class II loading and presentation to T cells [117]. Some proteins, however, are not easily unfolded for further processing by endolysosomal cathepsins. We have shown that the expression of Gamma-IFN-inducible Lysosomal Thiol reductase (GILT) is essential for the proper processing of disulfide containing Ags and peptides inside the cells [118–120]. GILT reduces disulfide bonds, allowing proteins to be unfolded and then processed by acidic proteases [121]. Removal of CLIP and formation of stable class II-peptide complexes is mediated by a nonclassical class II protein, HLA-DM [122, 123]. Once peptide is bound to a class II protein, the HLA class II-peptide complex is transported to the cell surface for presentation to CD4+ T cells (Figure 2).

**Figure 3:**
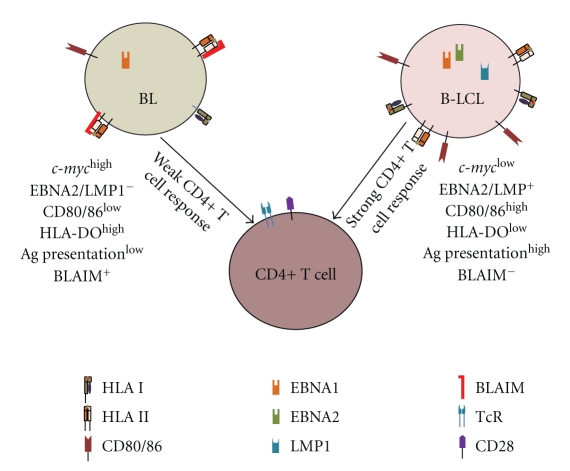
Comparison of BL and B-LCL highlighting the protein expression pattern of immune components that affects their abilities to stimulate CD4+ T cells. BL cells normally express EBNA1, but not EBNA2 and LMP1. When compared to B-LCL, BL cells express higher levels c-myc, HLA-DO and BLAIM and lower levels of costimulatory molecules (CD80/86) that differentially regulate Ag processing and presentation, resulting in poor CD4+ T cell recognition of BL.

BL cells express lower levels of costimulatory molecules (e.g., CD80 and CD86) that may modulate immune recognition via both class I and class II pathways (Figure 3; Table 1). HLA class II Ag presentation is also partially regulated by another nonclassical class II molecule, HLA-DO, which is primarily expressed by B lymphocytes [124]. Overexpression of HLA-DO molecules correlates to inhibition of HLA-DM activity, resulting in accumulation of cell-surface CLIP [125]. Studies have shown that the EBV positive BL cell line Raji expresses much higher levels of HLA-DO when compared to two EBV negative BL cell lines, Ramos, and BJAB [125, 126]. Elevated levels of HLA-DO in BL cells correlate with much higher levels of cell-surface CLIP and may interfere with peptide binding to class II molecules. This provides one mechanism by which BL may escape immune detection via the HLA class II pathway of Ag presentation (Figure 3; Table 1).

Accumulating evidence suggests that endogenous Ags are also processed and presented by the class II molecules for stimulation of CD4+ T cells [127, 128]. Study has also demonstrated the proteasome and TAP-dependent pathway of HLA class II Ag presentation for two influenza epitopes [129]. Among the proteasome independent pathways, only macroautophagy has been observed to deliver endogenous substrates to HLA class II [127, 130]. Endogenous Ag can also be processed and delivered by macroautophagy to HLA class II for presentation to and activation of CD4+ T cells [131, 132]. The involvement of macroautophagy in the presentation of EBNA1 to HLA class II may provide the means by which BL Ags could be presented to CD4+ T cells via the class II pathway. 

While EBNA1 is not optimally processed or presented through the HLA class I pathway, healthy EBV seropositive individuals do produce EBNA1-specific CD4+ T cells. Studies have demonstrated that under certain conditions EBNA1 specific T_h_ cells can recognize EBNA1 sensitized BL cells and mount an immune response, suggesting a role for HLA class II in the presentation of this Ag [133–135]. An EBNA1-specific CD4+ T-cell response was found to be required and sufficient to suppress tumor growth in a mouse model [136]. A possible role for CD4+ T cells in mounting an EBNA1-targeted response is further evidenced by the fact that children with eBL demonstrate a loss of EBNA1-specific, IFN-*γ*-secreting T cells [137, 138]. 

Various groups have demonstrated the capacity of B-LCL to efficiently present EBV-associated Ags to CD4+ T cells via HLA class II, but previously published results in our laboratory have revealed that BL cells are deficient in this ability [33, 139, 140]. We have demonstrated that BL express normal levels of HLA class II and the major components of the class II pathway, and that the expressed class II molecules were capable of binding peptide, but were incapable of activating CD4+ T cells (Figure 3). This was in contrast to B-LCL which functionally presented Ag to CD4+ T cells. The inability of BL to present Ag was overcome when the cells were incubated in a pH 5.5 buffer, and elutions of BL-associated molecules prevented functional Ag presentation by B-LCL (unpublished data). These findings suggest that BLAIM may inhibit Ag presentation by HLA class II molecules (Figure 3). Abnormalities of HLA class II protein function in BL cells also impair cellular and humoral immune responses, suggesting that insights into BL pathogenesis and immune escape should be considered for devising better immunotherapeutics against BL.

## 5. Conclusions

BL is a highly aggressive non-Hodgkin's lymphoma, found primarily in equatorial Africa and Papua New Guinea, but also observed with lesser frequency in other parts of the world. Cases of BL may vary in EBV association and clinical manifestation, but virtually all show *c-myc* translocation to an Ig locus and resultant overexpression. Although the relative contributions of EBV, HIV, and malaria to the development of BL remain largely unclear, we discussed a host of factors that may influence BL pathogenesis. Similarly, the mechanisms by which BL may evade class II-restricted immune recognition were discussed. Future studies may focus on molecular events that alter *c-myc* expression and immune-mediated elimination of BL. 

This paper suggests that the low immunogenicity of EBNA1 in EBV-positive BL is partially responsible for the poor CD8+ T cell response, but this does not account for the diminished CD4+ T-cell response. Because CD8+ T cells are incapable of clearing BL cells, HLA class II Ag presentation and immune recognition of BL should receive more attention. Recent studies also indicate that alternative pathways for Ag processing may exist which would allow for HLA class II presentation of BL Ags through macroautophagy or cross-presentation. However, ongoing research in our laboratory strongly suggests the existence of a BL-associated inhibitory molecule(s) which blocks CD4+ T-cell activation in the context of HLA class II molecules. Current studies are being conducted to isolate and characterize this inhibitory molecule and determine its mode of action. Successful identification of this molecule would open doors for novel therapeutic treatments of BL as well as shed light on immune evasion strategies that may be used by other malignancies.

 While currently used chemotherapy and chemoimmunotherapy treatment regimens for BL have achieved high survival rates in both adults and children, research in this area remains vitally important for several reasons: (a) adults show lower response rates than children, (b) adults are less tolerant of the treatment-associated toxicities and immunodeficiency, (c) HIV-associated BL patients suffer more severe mucositis and infections as a result of treatment, and (d) combination approaches are effective at improving survival rates, but the use of rituximab in HIV patients remains controversial. Factors that disrupt immune recognition demand further investigation for developing novel immunotherapies for better treatment and improved quality of life for the BL patients.

## Figures and Tables

**Table 1:** Factors may contribute to the defects in immune recognition of BL cells.

Factor	Cells Affected	Function(s)/defect(s) in Immune Evasion
EBNA1	>90% eBL	Negative regulation of own mRNA translation [64]
	5–10% sBL	Internal gly-ala repeat blocks proteasomal degradation and consequent
	40% HIV associated BL	HLA class I presentation [63]

C-myc	All BL	Downregulation of HLA class I [30]
		Antagonism of NF-*κ*B pathway
		Negative regulation of STAT1 signaling
		Impairment of interferon response [59]
		Downregulation of accessory molecules important in immune response: LFA-1, LFA-3, ICAM-1, and TAP [86–90]

HLA class I	All BL	Down-regulated in BL [71–74]
		Downregulation of CD80/86 in BL decreases HLA class I signaling

HLA class II	All BL	Downregulation of CD80/86 in BL decreases HLA class II signaling
		Upregulation of HLA-DO causes decrease in formation of class
		II/peptide complexes [91, 92] BLAIM may impair functional Ag
		presentation by HLA class II (unpublished data).

## Abbreviations

BL: Burkitt lymphoma
NHL: Non-Hodgkin's lymphoma
HL: Hodgkin's lymphoma
DLBCL: Diffused large B cell lymphoma
B-LCL: B-lymphoblastoid cell line
EBV: Epstein-Barr virus
HIV: Human immunodeficiency virus
EBNA: Epstein-Barr virus nuclear antigen
LMP: Latent membrane protein
BLAIM: BL-associated inhibitory molecules
HLA: Human leukocyte antigen
CLIP: Class II-associated invariant chain peptide
CTL: Cytotoxic T lymphocyte.
